# Heterotopic ossification in lymph node metastasis after rectal cancer resection: a case report and literature review

**DOI:** 10.1186/s12957-020-02098-x

**Published:** 2021-01-02

**Authors:** Hideki Nagano, Tamotsu Togawa, Takeshi Watanabe, Kenji Ohnishi, Toshihisa Kimura, Atsushi Iida, Sakon Noriki, Yoshiaki Imamura, Yasunori Sato, Takanori Goi

**Affiliations:** 1 Department of Surgery, National Hospital Organization Tsuruga Medical Center, 33-1, Sakuragaoka Tsuruga, Fukui, 914-0195 Japan; 2 Department of Surgery, Municipal Tsuruga Hospital, 1-6-60, Mishima-cho, Tsuruga, Fukui, 914-8502 Japan; 3 Division of Surgical Pathology, University of Fukui Hospital, 23-3 Matsuokashimoaizuki, Eiheiji-cho, Yoshida-gun, Fukui, 910-1193 Japan; 4 Institute of Medical, Pharmaceutical and Health Sciences, Faculty of Medicine, Kanazawa University, 13-1 Takara-machi, Kanazawa, Ishikawa 920-8641 Japan; 5 First Department of Surgery, Faculty of Medicine, University of Fukui, 23-3, Matsuokashimoaizuki, Eiheiji-cho, Yoshida-gun, Fukui, 910-1193 Japan

**Keywords:** Heterotopic ossification, Rectal cancer, Lymph node, Epithelial-mesenchymal transition, Osteoblast-like cell, BMP-2, TGF-β1, Gli2

## Abstract

**Background:**

Heterotopic ossification (HO) is the formation of osseous tissue outside the skeleton. HO in malignant tumors of the digestive tract is extremely rare, as is ossification in metastatic lesions from HO-negative digestive tract tumors. Regarding the pathogenesis of HO, two theories have been proposed. The first is that the osteoblastic metaplasia of tumor cells (driven by the epithelial-mesenchymal transition, EMT) results in HO, and the second is that factors secreted by cancer cells lead to the metaplasia of stromal pluripotent cells into osteoblasts. However, the osteogenic mechanisms remain unclear.

**Case presentation:**

An 83-year-old Japanese woman underwent low anterior rectal resection for rectal cancer before presentation at our institution, in June 2018. The final diagnosis was stage IIB rectal adenocarcinoma (T4aN0M0). Histological examination did not reveal HO in the primary tumor. Thirteen months after the operation, a solitary metastatic lesion in the brain 20 mm in size and a solitary metastatic lesion in a right axillary lymph node 20 mm in size were diagnosed. The patient was treated with gamma-knife therapy for the brain metastasis. One month later, she was referred to our institution. She underwent lymph node resection. Histological examination revealed that most portions of the affected lymph node were occupied by metastatic tumor cells and that central necrosis and four small ossified lesions without an osteoblast-like cell rim were present in the peripheral region. Immunohistochemical analysis showed tumor cells positive for BMP-2, osteonectin, osteocalcin, AE1/AE3, TGF-β1, Gli2, Smad2/3, and CDX2 and negative for nestin, CD56, and CK7.

**Conclusion:**

This is the first English case report of HO in a metachronous metastatic lymph node after the curative resection of HO-negative rectal cancer. Unlike HO lesions in past reports, the HO lesion did not show peripheral osteoblast-like cells, and the immunohistochemical findings indicated that the present case resulted from the EMT.

## Background

Heterotopic ossification (HO) is bone tissue formation outside the skeleton; the lesion may be benign or malignant [1, 2]. This phenomenon has been reported in tumors of various organs, including the lung, breast, thyroid, parotid, pancreas, liver, and kidney [3–7]. There are rare reports of HO in the gastrointestinal tract, with the greatest prevalence in the colon and rectum; however, to date, there have been only 18 English reports of HO cases derived from primary rectal cancer. The presence of HO lesions in both primary rectal cancer and metastatic lymph nodes has been reported in only one case [8]. Although there are a few reports of HO lesions in sites of recurrence, such as the lungs or laparotomy sites [3, 9], HO in metachronous metastatic lymph nodes from rectal carcinoma has not been reported in the English literature, to the best of our knowledge. Herein, we report a case of HO in a metachronous metastatic lymph node in the right axillary region after curative surgery for rectal cancer without HO and treatment by gamma-knife therapy for metachronous brain metastasis without HO. The purpose of this article is to highlight the histopathological and immunohistochemical findings of HO in the metastatic lymph node and consider the pathogenesis of HO in the present case.

## Case presentation

**Fig. 1:**
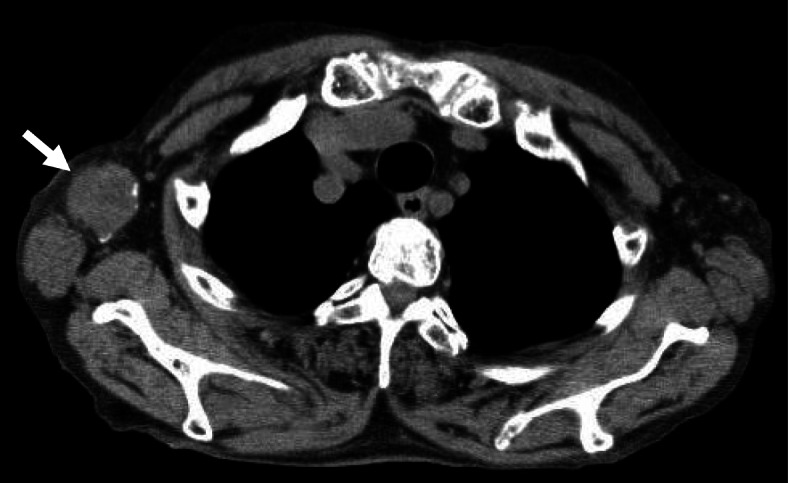
Findings of CT performed in October 2019. A solitary swollen lymph node measuring 37 × 33 mm in size with small high-density spots in the peripheral region was observed in the right axillary region (arrow)

An 83-year-old Japanese woman underwent laparoscopic low anterior rectal resection accompanied by bilateral lymph node dissection for rectal cancer without neoadjuvant chemotherapy at a domestic general hospital in June 2018. Preoperative diagnostic imaging that included chest to pelvic computed tomography (CT) showed no high-density spots in the rectal tumor and no distant metastasis. The histopathological diagnosis of the tumor was rectal adenocarcinoma, T4aN0M0, stage IIB, consisting of high-to-low differentiation grade. After the surgery, she presented with anastomotic leakage and underwent temporary ileostomy; the leakage healed 3 months after the rectal resection. Considering the tumor stage and the patient’s advanced age, adjuvant chemotherapy was not applied. She underwent closure of the ileostoma in January 2019. In July 2019, she was diagnosed with a metastatic brain lesion 20 mm in size on the left prefrontal cortex with cerebral edema and a metastatic right axillary lymph node enlarged to 20 mm in size without small high-density spots by CT (Supplement 1). She was treated with gamma-knife therapy with a total of 20 Gy of irradiation, and the metastatic brain tumor and cerebral edema disappeared. Because of the recurrence, the previous surgeon reconsidered the policy for adjuvant chemotherapy and applied chemotherapy consisting of oral S1 (composed of tegafur and gimeracil, the latter being an inhibitor of dihydropyrimidine dehydrogenase) for the metastatic right axillary lymph node in August 2019. The patient moved to the city of Tsuruga with her family and was referred to the Tsuruga Medical Center in October 2019. She presented a round-shaped, swollen solitary lymph node in the right axillary region that was not fixed to the adjacent structures. The serum levels of epithelial tumor markers, such as carcinoembryonic antigen (CEA; 12.5 ng/ml), carbohydrate antigen 19-9 (CA19-9; 187.7 U/ml), and cancer antigen 125 (CA125; 41 U/ml), were elevated. Plain CT (64-row multidetector CT, Aquilion, TSX-101A, Toshiba, Japan) showed a solitary swollen lymph node in the right axillary region, 37 × 33 mm, with small high-density spots in the peripheral region (Fig. 1). We performed lymph node resection under the tentative diagnosis of right axillary lymph node metastasis with calcified deposits from rectal cancer in November 2019. The patient showed an uneventful recovery, and chemotherapy consisting of tegafur-uracil/calcium folinate (UFT/UZEL) was initiated 10 days after the operation; she was discharged on the thirteenth postoperative day. She was admitted to the internal medicine section of our hospital for acute pneumonia in January 2020. Chest CT revealed acute pneumonia and swollen right axillary lymph nodes (42 × 34 mm and 24 × 18 mm in size) without high-density spots (Supplement 2A, B). The pneumonia progressed rapidly, and the patient died 4 days later. An autopsy was not carried out because her family did not provide consent.

### Pathological findings

#### Gross pathology of the primary rectal tumor

**Fig. 2:**
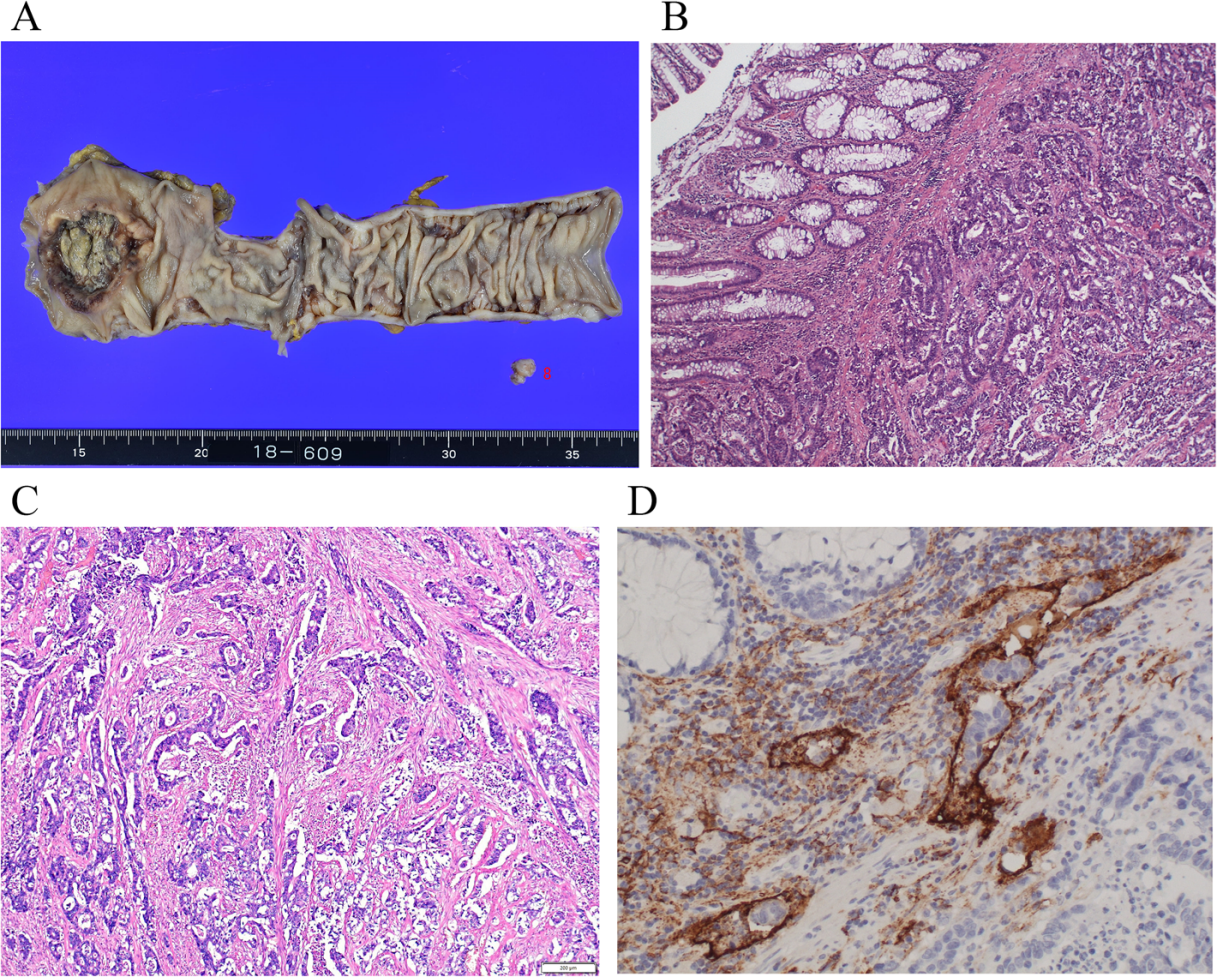
**a** Gross appearance of resected rectal cancer. The resected specimen contains a polypoid tumor accompanied by an ulcer with a fungating polypoid component, measuring 48 × 46 mm. **b** Microscopy of the primary tumor. The primary tumor was mainly composed of moderately differentiated adenocarcinoma. **c** In a portion of the tumor, poorly differentiated carcinoma components were found. **d** Scattered lymphatic invasion was found (immunostaining by D2-40)

The rectal tumor was cut and opened. The resected specimen consisted of a polypoid tumor accompanied by an ulcer with a fungating polypoid mass, measuring 48 × 46 mm and occupying two-thirds of the circumference of the lower to middle rectum (Fig. 2a). The distal margin was 10 mm.

#### Microscopy of the primary tumor

The primary tumor was mainly composed of moderately differentiated adenocarcinoma, and in a portion of the tumor, well-differentiated adenocarcinoma and poorly differentiated carcinoma components were found (Fig. 2 b and c). Scattered lymphatic and venous invasion (Fig. 2d) and perineural invasion were noted. Ossified areas were not seen in the primary tumor. In the lymph nodes resected with the primary rectal tumor, metastasis was not found.

#### Pathological findings of the axillary lymph node

**Fig. 3:**
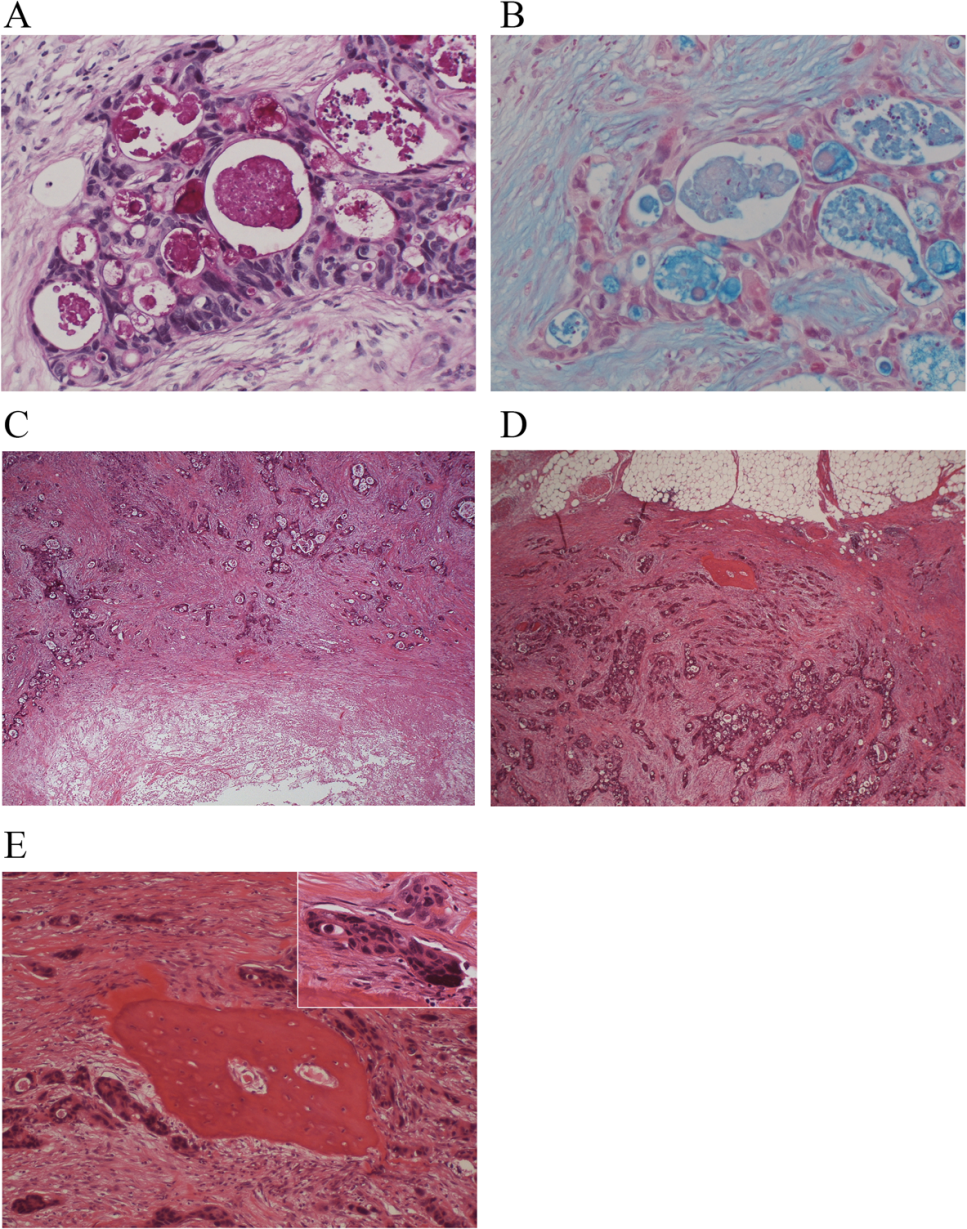
Microscopic findings of the axillary lymph node. **a** Metastatic tumor cells contained mucin as indicated by D-PAS staining; however, mucin retention in the stroma was not observed. **b** The tumor cells were also found positive for mucin by alcian blue staining. **c** Necrosis was observed in the central region of the node, and the metastasized tumor cells were directly adjacent. **d** In the peripheral region of the lymph node, four small ossified lesions were found, and the metastasized tumor cells were directly adjacent to ossified nests (arrow) (× 40). **e** Osteocytes were observed inside the osseous matrix; however, osteoblast-like cells were not found around the HO lesion (× 100). The upper right is a magnified view of the tumor cells beside the HO lesion

The axillary lymph node contained metastatic adenocarcinoma that was D-PAS and alcian blue positive; the primary lesion also stained positive, but there was no retention of mucin in the interstitial tissue (Fig. 3a, b). Most portions of the lymph node were occupied by metastatic tumor cells. Extensive necrosis was observed in the central region of the node and was accompanied by interstitial fibrosis; the metastasized tumor cells were directly adjacent to necrosis (Fig. 3c). The metastatic adenocarcinoma was a poorly to moderately differentiated adenocarcinoma with a trabecular and cribriform pattern. In the peripheral region of the lymph node, four small ossified lesions were found, and the metastasized tumor cells were directly adjacent to ossified nests (Fig. 3d). Osteocytes were observed inside the osseous matrix; however, osteoblast-like cells were not found around the heterotopic osseous lesion (Fig. 3e), and hematopoietic fatty marrow was not observed.

#### Azan staining and immunohistochemical staining of the axillary lymph node (details of the antibodies used are provided in Supplement 3)

**Fig. 4:**
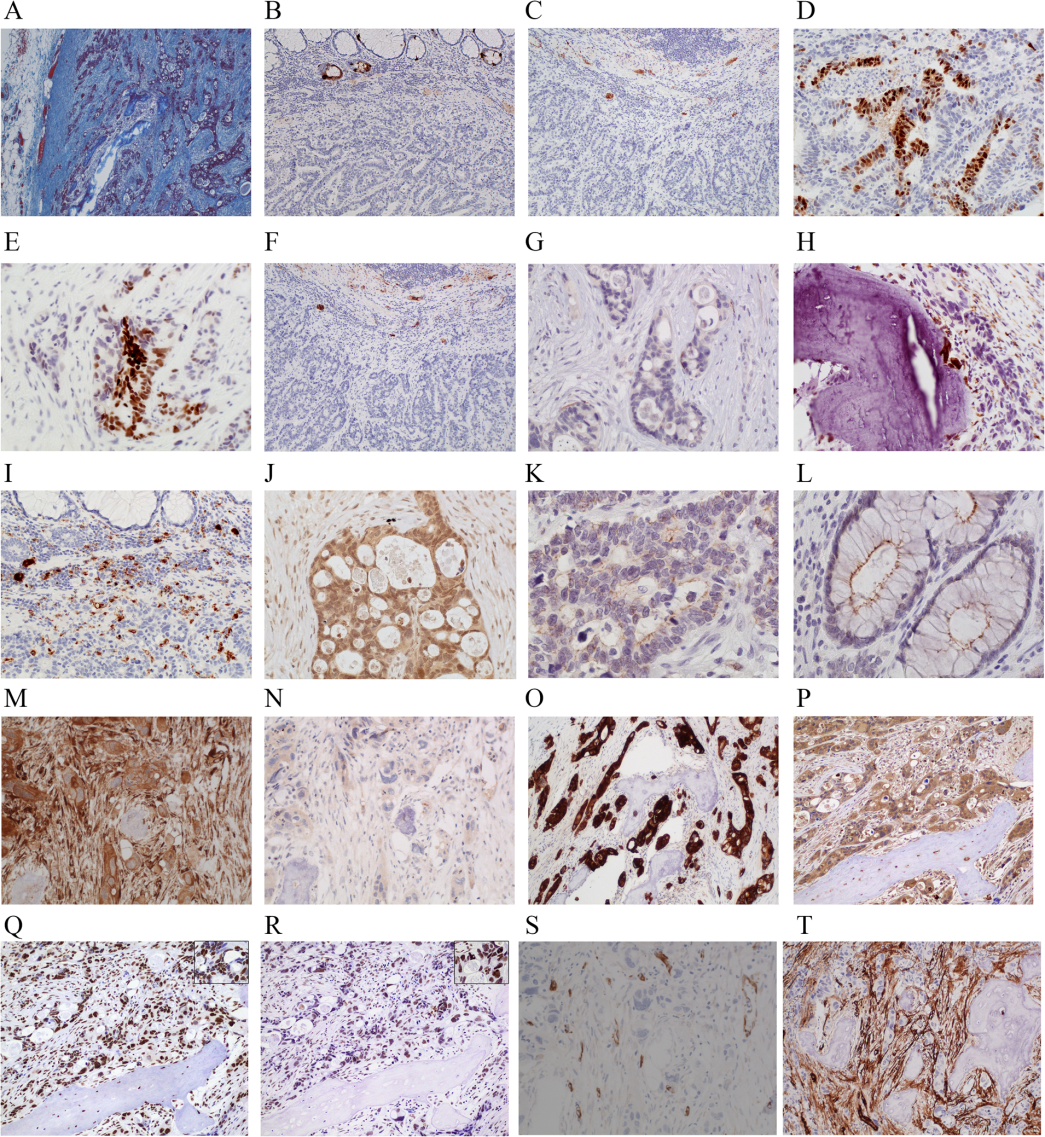
Findings of Azan staining and immunohistochemical staining of the axillary lymph node. **a** Azan staining showed that the metastatic tumor lesion was collagen rich (× 40). **b** Immunohistochemical staining using an antibody for chromogranin A showed negative staining in the metastatic tumor lesion as well as in the primary lesion. **c** The tumor cells also showed negative staining for synaptophysin. **d** The primary tumor cells showed positive staining for CDX2 in the nucleus (× 200). **e** The tumor cells in the lymph node also showed positive staining for CDX2 (× 200). **f**, **g** The tumor cells in the lymph node were negative for osteoblast marker CD56 (**f**) and breast cancer marker CK7 (**g**). **h** Cells positive for the macrophage cell marker CD68 were observed around the osseous lesion. **i** CD68-positive cells were also seen in the primary tumor. **j** The tumor cells showed BMP-2 overexpression in the lymph node (× 400). **k**, **l** BMP-2 expression was weakly positive in the cell membrane on the lumen side of the primary tumor (**k**: × 400) and adjacent normal mucosa (**l**: × 400). **m**, **n** Osteonectin expression was strongly positive (**m**: × 200) and osteocalcin expression was weakly positive (**n**: × 200) in the tumor cells. **o**, **p** The tumor cells expressed cytokeratin (AE1/AE3) (**o**: × 200) and TGF-β1 (**p**: × 200; *HO tissue). In the HO lesion, cytokeratin-positive osteocytes were observed (**o**: arrows). **q**, **r** Gli2 (**q**: × 200) and pSmad2/3 (**r**: × 200) showed overexpression in the nucleus of the tumor cells (*HO tissue). The upper right of **q** and **r** are magnified views (× 400). **s** The tumor cells showed no nestin expression (× 200). **t** Fibroblasts surrounding the tumor cells showed alpha-smooth muscle actin (α-SMA) expression (× 100)

Staining with Azan revealed collagen-rich stroma in the metastatic lesion (Fig. 4a). Immunostaining for neuroendocrine markers such as chromogranin A (Fig. 4b) and synaptophysin (Fig. 4c) showed negative staining in the metastatic lesion as well as in the primary lesion. (The immunostaining protocol is described in Supplement 4.) Positive staining for caudal-type homeobox 2 (CDX2) was observed in both the primary tumor (Fig. 4d) and metastatic lymph node (Fig. 4e), suggesting that the lymph node lesion was a metastasis of rectal cancer. The tumor cells were negative for CD56 (an osteoblast marker) (Fig. 4f) and CK7 (Fig. 4g). Cells positive for CD68 (a macrophage cell marker) were observed around the osseous lesion (Fig. 4h). CD68-positive cells were also seen in the primary tumor (Fig. 4i).

Bone morphogenetic protein 2 (BMP-2) was observed to be weakly positive in the tumor cells in the metastatic lymph node (Fig. 4j), the luminal membrane of the primary tumor (Fig. 4k), and adjacent normal mucosa (Fig. 4l); the primary tumor was judged to be negative for BMP-2. The metastatic tumor cells were also positive for osteonectin (Fig. 4m), osteocalcin (Fig. 4n), cytokeratin AE1/AE3 (Fig. 4o), transforming growth factor-β1 (TGF-β1) (Fig. 4p), transcription factor glioma-associated oncogene protein 2 (Gli2) (Fig. 4q), and phosphorylated Smad2/3 (pSmad2/3) (Fig. 4r) but were negative for nestin (Fig. 4s), murine double minute 2 (MDM2, data not shown), and cluster of differentiation 34 (CD34, data not shown). Figure 4o showed AE1/AE3-positive osteocytes in the HO lesion. BMP-2 and TGF-β1 expression was observed in the cytoplasm of the tumor cells, whereas Gli2 and pSmad2/3 expression was observed in the nucleus of the tumor cells. The expression of alpha-smooth muscle actin (α-SMA) was observed in fibroblasts surrounding the osseous nests (Fig. 4t).

## Discussion

HO is the formation of osseous tissue outside the skeleton. This phenomenon has been observed in the liver, breast, skin, kidney, lung, thyroid, pancreas, and so on [3–7]. According to 2 review articles, the most common sites of HO in the gastrointestinal tract are the colon and rectum [10, 11]. The first 2 cases of rectal adenocarcinoma with bone formation were described by Hasegawa [12] in a report published in German in 1923. Since Dukes [13] provided the first report in the English literature of heterotopic bone formation in rectal carcinoma, describing 2 cases, in 1939, there have been, to the best of our knowledge, 18 reported cases of HO in primary rectal carcinoma [6, 7, 10, 11, 13–25] written in English. Only one of these cases involved an HO lesion in a metastatic lymph node [8] (Table 1). Additionally, Goswami [2] reported a case in which a metastatic lymph node with HO was simultaneously dissected with the primary rectal carcinoma lesion, which was without HO. However, HO in a lymph node with metachronous metastasis associated with rectal cancer without HO has not yet been reported. Only three cases, including the present case, of HO in a metastatic lymph node from rectal carcinoma have been reported in English to date [2, 8]. While the two previous cases involved synchronous HO in the regional metastatic lymph nodes, the present case involved metachronous HO in the distant lymph node. Moreover, the prior two cases involved an osteoblast-like cell rim, which was absent in our case.

**Table 1:** Reported cases of primary rectal cancer with heterotopic ossification [7, 8, 10, 11, 13–25]

Case	Reference		Age	Gender	LN metastasis present	Ossification in LN metastasis	Metastasis to other organ	Ossification in metastases	osteoblast-like cell
1	1939	Dukes [13]	69	M	NA		NA		NA
2	1939	Dukes [13]	32	F	-		NA		+
3	1951	Christie [14]	44	F	+	NA	NA		+
4	1962	Urbanke [15]	55	F	NA		NA		+
5	1988	Byard [16]	72	M	NA		NA		NA
6	1992	Ansari [10]	54	F	-		+	NA	+
7	1993	Pai [17]	46	M	+	-	NA		NA
8	1996	Haque [11]	78	M	NA		NA		+
9	1997	Beauchamp [18]	64	M	+	NA	NA		+
10	2003	Kypson [19]	38	F	-		-		NA
11	2004	Matsumoto [20]	67	M	+	-	-		NA
12	2004	Szumilo [21]	79	M	+	-	NA		+
13	2005	Al-Maghrabi [22]	90	F	+	NA	-		+
14	2010	Nagao [8]	46	M	+	+	NA		+
15	2011	Badmos [7]	48	M	-		NA		+
16	2015	Smajda [23]	29	F	+	NA	+	NA	NA
17	2016	Shimazaki [24]	57	M	+	NA	NA		+
18	2017	Liu [25]	76	M	+	-	-		+

*NA* not available

Regarding the pathogenesis of HO, two hypotheses have been proposed: The first is that the epithelial-mesenchymal transition (EMT) upregulates tumor cell production of osteogenic factors and induces osteoblast-like transformation, which stimulates uncommitted mesenchymal stromal cells toward osteoblastic differentiation and contributes to heterotopic bone formation [10, 26, 27]. The second is that a diversity of BMP-secreting tumor cells stimulates the osteogenic metaplasia of undifferentiated stromal mesenchymal cells or fibroblasts into osteoprogenitor cells. The second hypothesis seems to account for most cases [15, 28–30]. Among the 18 reported cases of primary rectal cancer with HO, osteoblast-like cells were present in 12 cases; in the remaining 6 cases, their presence or absence was not described. However, in the present case, neither osteoblast-like cells nor pluripotent stromal cells were observed around the osseous matrix, and the carcinoma cells showed osteonectin, osteocalcin, BMP-2, TGF-β1, and Gli2 expression.

Osteonectin was strongly positive, and osteocalcin was weakly positive in the tumor cells. These proteins are noncollagenous components of the bone secreted by osteoblasts.

BMP-2 is associated with osteogenic activity and can stimulate bone formation [31], actively inducing the osteoblastic differentiation of both immature osteoblasts and less committed cells; its overexpression in rectal adenocarcinoma cells was first reported by Kypson et al. [19]. BMPs are a group of TGF molecules that have the ability to induce bone and cartilage formation, and TGF-β1 activity strongly enhances BMP-2 activity [32]. TGF-β is known to induce the EMT, whereby epithelial tumor cells acquire an invasive, mesenchymal-like phenotype [33]. The presence of TGF-β1 in tumor cells demonstrated by immunohistochemistry suggests that tumor cells secrete this factor into the microenvironment, which might, much like BMP-2, stimulate pluripotent stromal cells or fibroblasts to transform into osteocytes [1]. The expression of α-SMA was observed in fibroblasts surrounding the osseous nests. TGF-β1 can induce α-SMA expression in fibroblasts, which further implicates the occurrence of TGF-β1 activity in the microenvironment [34–36]. TGF-β1 activates Smad (through the Smad pathway) and p38MAPK (through the non-Smad-dependent pathway). These two factors converge at runt-related transcription factor 2 (Runx2), which is the most upstream transcription factor essential for osteoblast differentiation, to induce bone formation [37]. Gli2, a downstream molecule of TGF-β signaling involved in driving cancer progression toward metastasis, provides cancer cells with a more aggressive phenotype and metastatic ability [33]. In addition, Gli2 can stimulate the production of BMP [38, 39]. Unlike in past reports, in the present case, osteoblast-like cells or pluripotent cells were not observed around the HO lesion, and the metastatic adenocarcinoma cells themselves produced every ossification factor and bone material and served the role of osteoblasts. This process seems to resemble the pathogenesis of fibrous dysplasia, a skeletal condition in which normal bone is replaced by poorly organized bone and fibrous tissue with little to no osteoblastic rim [40].

Regarding the intermediate filaments, generally, it is thought that tumors with intermediate filament conversion, such as vimentin-expressing tumors, have high malignancy. In the present case, the tumor cells were positive for AE1/AE3 and negative for nestin, which is an intermediate filament protein abundant in embryonic stem-derived progenitor cells that can differentiate into cells of various lineages, including mesodermal cells [41], and be used for the analysis of osteoblasts. These results suggest that the tumor cells might have obtained the ability to form bone without intermediate filament conversion; however, the observed negativity for nestin alone does not seem sufficient to confirm or negate this possibility. In the HO lesion, cytokeratin-positive osteocytes were also observed (Fig. 4o), and the results suggest the possibility that the tumor cells induce the metaplasia of fibroblasts to osteocytes via the EMT.

**Fig. 5:**
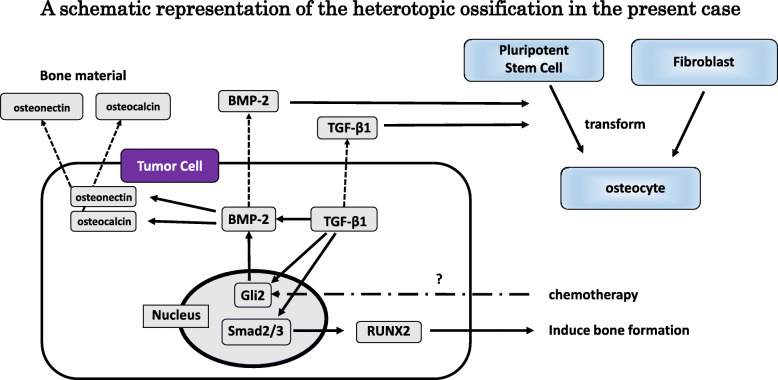
A schematic representation of the HO in the present case. Although the primary tumor did not produce BMP-2 nor show HO, the metastasized tumor cells acquired the ability to express BMP-2 and TGF-β1. TGF-β1 stimulated the expression of BMP-2 and Gli2, and Gli2 stimulated the expression of BMP-2. BMP-2 induced the production of bone material, such as osteonectin or osteocalcin. BMP-2 and TGF-β1 were secreted outside the tumor cells and transformed fibroblasts and, probably, pluripotent stem cells into osteocytes. In addition, Gli2 stimulated the Smad pathway in the nucleus and induced bone formation through the RUNX2 pathway. Chemotherapy might contribute to Gli2 overexpression by activating the Hedgehog pathway. These findings provide evidence that HO can occur independently, without the transformation of pluripotent stem cells into osteoblast-like cells, and that the present case resulted from osteogenesis via the EMT. In contrast, most previously reported cases resulted from the metaplasia of stromal cells into osteoblasts

**Fig. 6:**
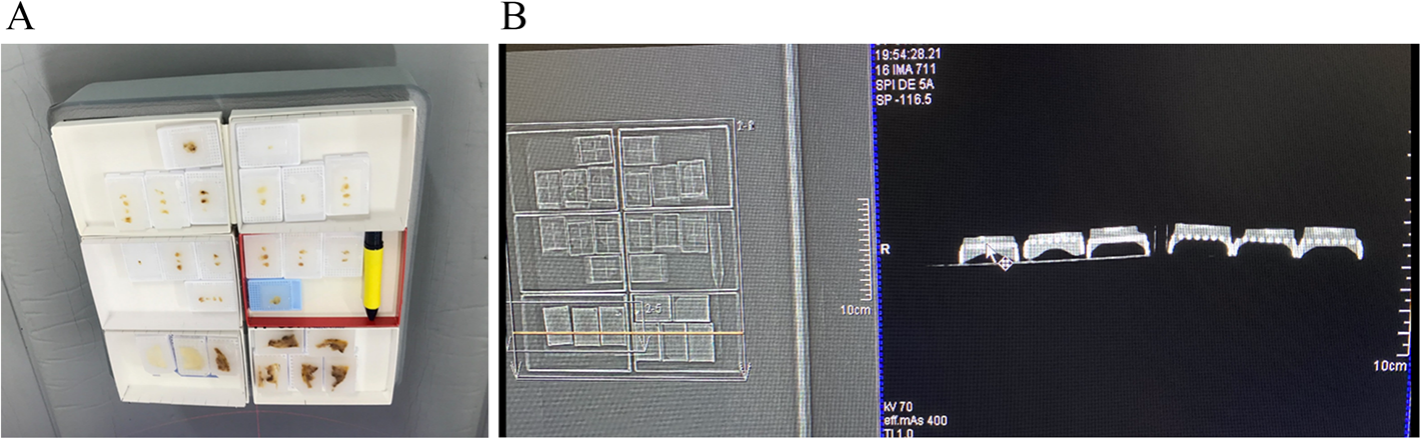
CT findings of the paraffin block of the rectal cancer, regional lymph nodes, and axillary lymph node. **a** The specimen blocks were set on the CT. Two left lower blocks, outlined in red, are of axillary lymph node with ossification. **b** The yellow horizontal line of the left image indicates the scan level of the CT. The left-hand side of the right image shows the CT of the axillary lymph node, and a minute high-density spot was apparent in the left specimen (arrow); however, not such spot was observed in the primary tumor specimen

Mucin, necrosis, and desmoplastic stroma are considered relevant factors of HO [9, 11, 13, 42]. In the present case, the tumor cells contacted necrotic lesion directly, but mucin or desmoplastic stroma was not observed in the interstitial tissue. Minute ossified lesions were in contact with tumor cells but were not in direct contact with necrotic tissue. The influence of necrosis on HO in this case is unclear. Smajda et al. [23] reported HO in association with FOLFOX6 administration followed by a combination of capecitabine and radiotherapy. In the present case, at the time of diagnosis of axillary lymph node metastasis in July 2019, HO was not detected by CT, and administration of an anticancer agent (S1) was begun following diagnosis HO subsequently appeared in the peripheral portion of the lymph node in October 2019. S1 consisted of tegafur, a 5-fluorouracil (5FU) prodrug. Yu et al. [43] reported that 5FU treatment elevated the expression of hedgehog target genes GLI1 and GLI2 in gastric cancer. The hedgehog pathway plays a role in bone formation as it allows the differentiation of mesenchymal progenitor cells into osteoblast cells [44]. In our case, it is possible that chemotherapy-induced activation of the hedgehog pathway contributed to the overexpression of Gli2 and resulted in HO. Figure 5 shows a schematic representation of the HO in the present case. The HO lesions observed in this case were < 1 mm and identified mainly by CT imaging. To definitively diagnose the presence or absence of HO, investigation of 1-mm slices of the entire primary rectal tumor is necessary; however, this was technically impossible in our case. We conducted a CT scan of the stored gross specimens of the primary tumor and axillary lymph node. The scan revealed that the primary tumor was negative for high-density spots, whereas the lymph node was positive (Fig. 6). Based on the negative BMP-2 findings for the primary lesion, the preoperative CT of the primary tumor, and the CT scans of the specimens, we concluded that HO was not evident in the primary tumor. Metastasis to a lymph node in the axillary region occurred, but the positive reaction for CDX2 and the negative reaction for CK7 indicated that the metastatic foci were from rectal cancer and not from unknown breast cancer.

Generally, there are two pathogenic mechanisms of ossification: membranous ossification or endochondral ossification under normal conditions and HO under pathological conditions. With respect to HO, it seems that there are many pathogenic mechanisms that have yet to be elucidated.

Axillary lymph node metastases recurred 2 months after resection of the right axillary metastatic lymph node. At the time of recurrence to the same region, HO was not detected by CT. Although a pathological diagnosis was not obtained from the recurrent axillary lymph nodes, HO might not always occur even if similar adenocarcinoma cells metastasize, and there might be an optimum condition for HO considering the secretion of factors by tumor cells.

## Conclusions

We report the first case of HO in a metachronous metastatic lymph node from rectal cancer without HO. Unlike in past reports, in the present case, neither osteoblast-like cells nor pluripotent cells were observed around the HO lesions, and the metastatic adenocarcinoma cells produced ossification factors and bone material. These findings indicated that the present case resulted from osteogenesis via the EMT; in contrast, most previously reported cases resulted from the metaplasia of stromal cells into osteoblasts. The documentation and detailed analysis of additional cases are necessary.

## Supplementary Information

**Additional file 1: Supplement 1.** CT findings of the recurrent right axillary lymph node in June 2019. A metastatic lymph node enlarged to 20 mm in size without small high-density spots was found (arrow); then, administration of an anticancer agent (S1) was begun. **Supplement 2.** Findings of CT performed in January 2020. Axillary lymph node metastases recurred (42 × 34 mm and 24 × 18 mm in size; A: arrow, B: arrows) two months after resection of the right axillary metastatic lymph node. Despite the recurrence in the same region, high-density spots were not observed in the lymph nodes. **Supplement 3.** Details of the antibodies used in the present case. **Supplement 4.** The immunostaining protocol used in the present case.

## Data Availability

All data generated or analyzed during this study are included in this published article.

## Abbreviations

HO: Heterotopic ossification
CT: Computed tomography
CEA: Carcinoembryonic antigen
CA19-9: Carbohydrate antigen 19-9
CA125: Cancer antigen 125
UFT/UZEL: Tegafur uracil/calcium folinate
CDX2: Caudal-type homeobox 2
CD56: Cluster of differentiation 56
CK7: Cytokeratin 7
CD68: Cluster of differentiation 68
BMP-2: Bone morphogenetic protein-2
TGF-β1: Transforming growth factor-β1
Gli2: Transcription factor glioma-associated oncogene protein 2
pSmad2/3: Phosphorylated Smad2/3
MDM2: Murine double minute 2
CD34: Cluster of differentiation 34
α-SMA: Alpha smooth muscle actin
EMT: Epithelial-mesenchymal transition
MAPK: Mitogen-activated protein kinase
Runx2: Runt-related transcription factor 2
FOLFOX: Folic acid, fluorouracil, oxaliplatin
5FU: 5-Fluorouracil
