# Supplementary material for: Heterotopic ossification in lymph node metastasis after rectal cancer resection: a case report and literature review
Source: World J Surg Oncol. 2021 Jan 2;19:2. doi: 10.1186/s12957-020-02098-x (PMC7778818; doi:10.1186/s12957-020-02098-x)
Supplement: Supplementary file 1 — Additional file 1: Supplement 1. CT findings of the recurrent right axillary lymph node in June 2019. A metastatic lymph node enlarged to 20 mm in size without small high-density spots was found (arrow); then, administration of an anticancer agent (S1) was begun. Supplement 2. Findings of CT performed in January 2020. Axillary lymph node metastases recurred (42 × 34 mm and 24 × 18 mm in size; A: arrow, B: arrows) two months after resection of the right axillary metastatic lymph node. Despite the recurrence in the same region, high-density spots were not observed in the lymph nodes. Supplement 3. Details of the antibodies used in the present case. Supplement 4. The immunostaining protocol used in the present case. [file 12957_2020_2098_MOESM1_ESM.pptx]

## Slide 1
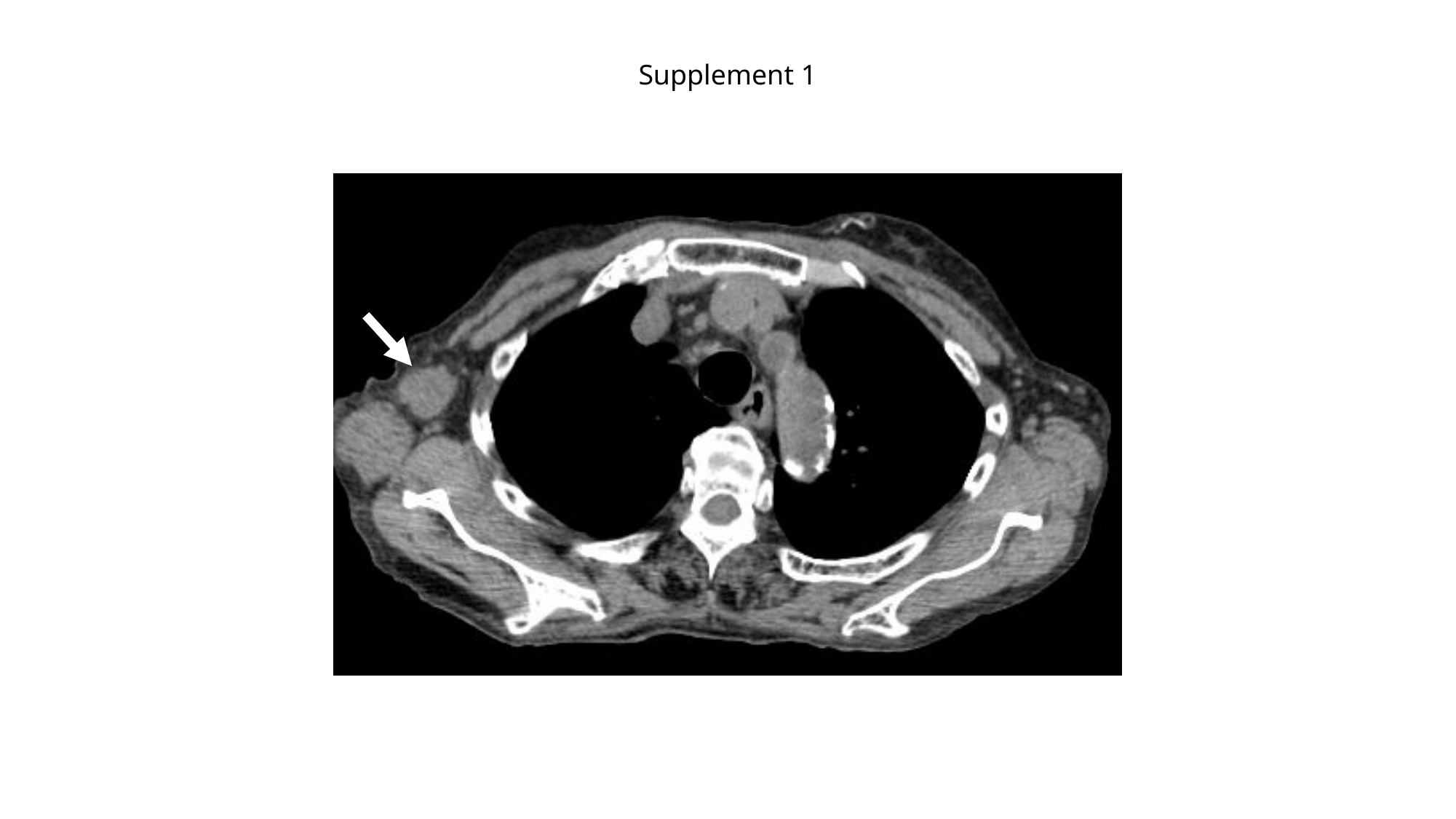

Supplement 1

## Slide 2
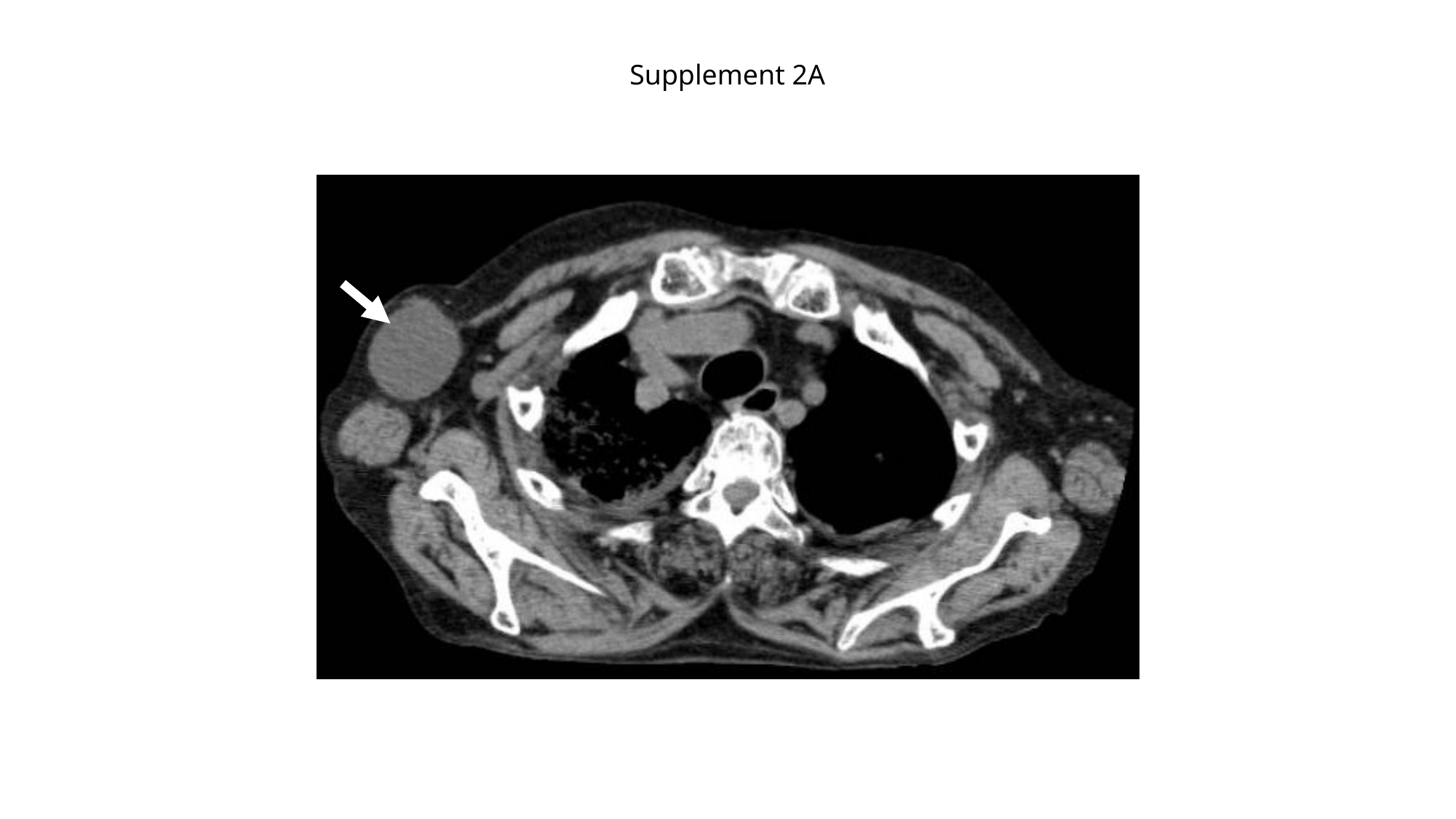

Supplement 2A

## Slide 3
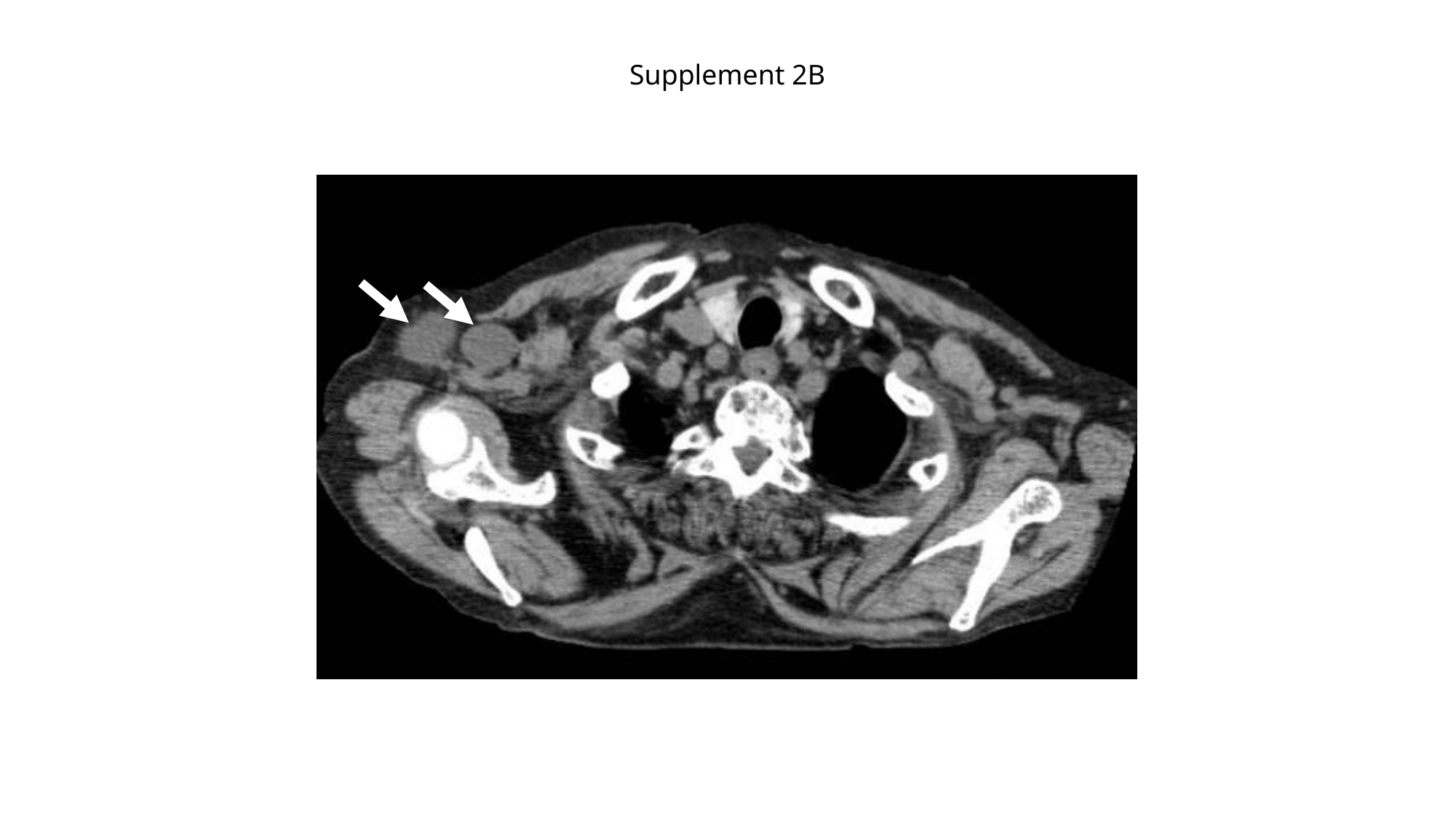

Supplement 2B

## Slide 4
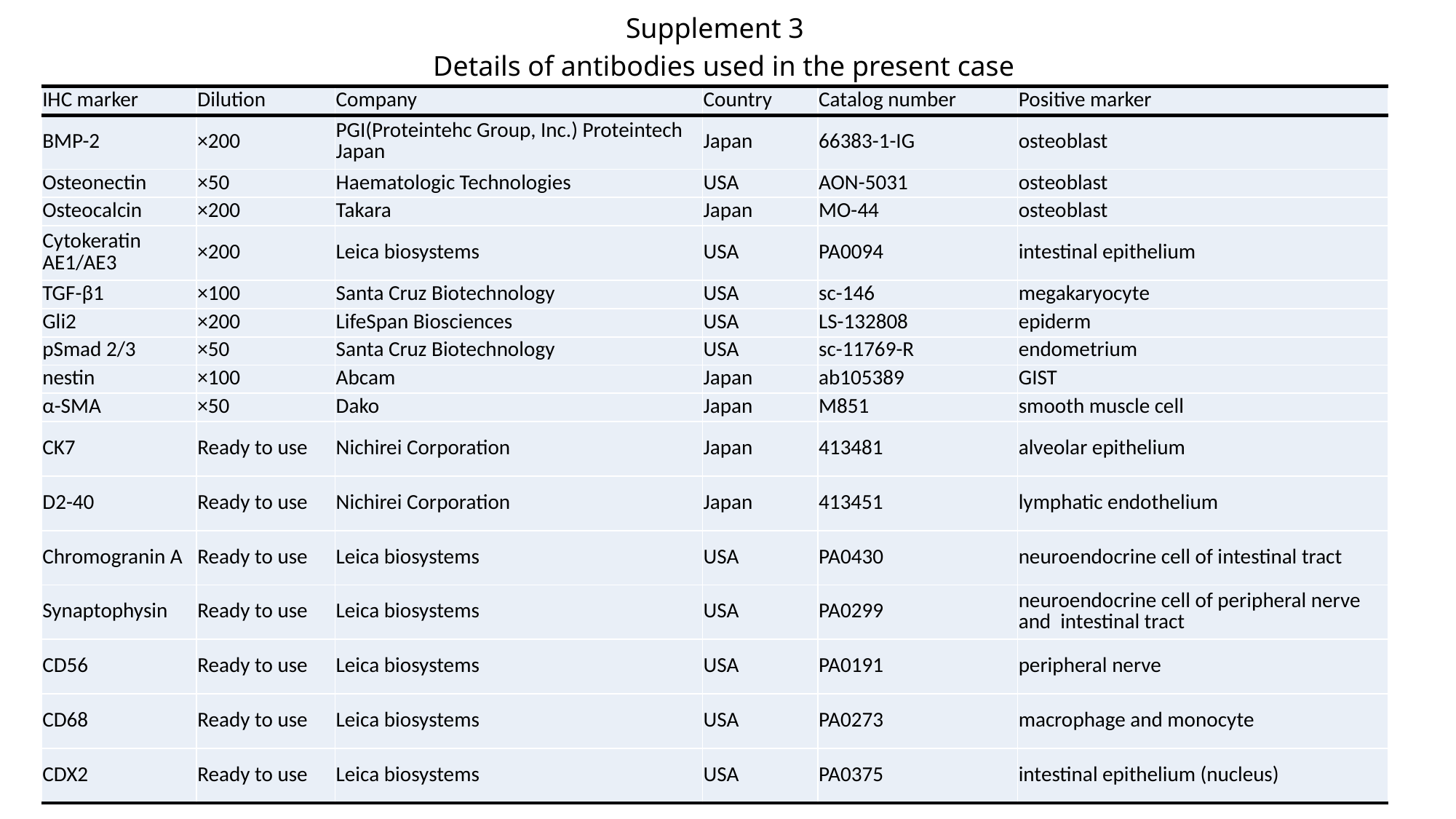

Supplement 3
Details of antibodies used in the present case
| IHC marker | Dilution | Company | Country | Catalog number | Positive marker |
| --- | --- | --- | --- | --- | --- |
| BMP-2 | ×200 | PGI(Proteintehc Group, Inc.) Proteintech Japan | Japan | 66383-1-IG | osteoblast |
| Osteonectin | ×50 | Haematologic Technologies | USA | AON-5031 | osteoblast |
| Osteocalcin | ×200 | Takara | Japan | MO-44 | osteoblast |
| Cytokeratin AE1/AE3 | ×200 | Leica biosystems | USA | PA0094 | intestinal epithelium |
| TGF-β1 | ×100 | Santa Cruz Biotechnology | USA | sc-146 | megakaryocyte |
| Gli2 | ×200 | LifeSpan Biosciences | USA | LS-132808 | epiderm |
| pSmad 2/3 | ×50 | Santa Cruz Biotechnology | USA | sc-11769-R | endometrium |
| nestin | ×100 | Abcam | Japan | ab105389 | GIST |
| α-SMA | ×50 | Dako | Japan | M851 | smooth muscle cell |
| CK7 | Ready to use | Nichirei Corporation | Japan | 413481 | alveolar epithelium |
| D2-40 | Ready to use | Nichirei Corporation | Japan | 413451 | lymphatic endothelium |
| Chromogranin A | Ready to use | Leica biosystems | USA | PA0430 | neuroendocrine cell of intestinal tract |
| Synaptophysin | Ready to use | Leica biosystems | USA | PA0299 | neuroendocrine cell of peripheral nerve and intestinal tract |
| CD56 | Ready to use | Leica biosystems | USA | PA0191 | peripheral nerve |
| CD68 | Ready to use | Leica biosystems | USA | PA0273 | macrophage and monocyte |
| CDX2 | Ready to use | Leica biosystems | USA | PA0375 | intestinal epithelium (nucleus) |

## Slide 5
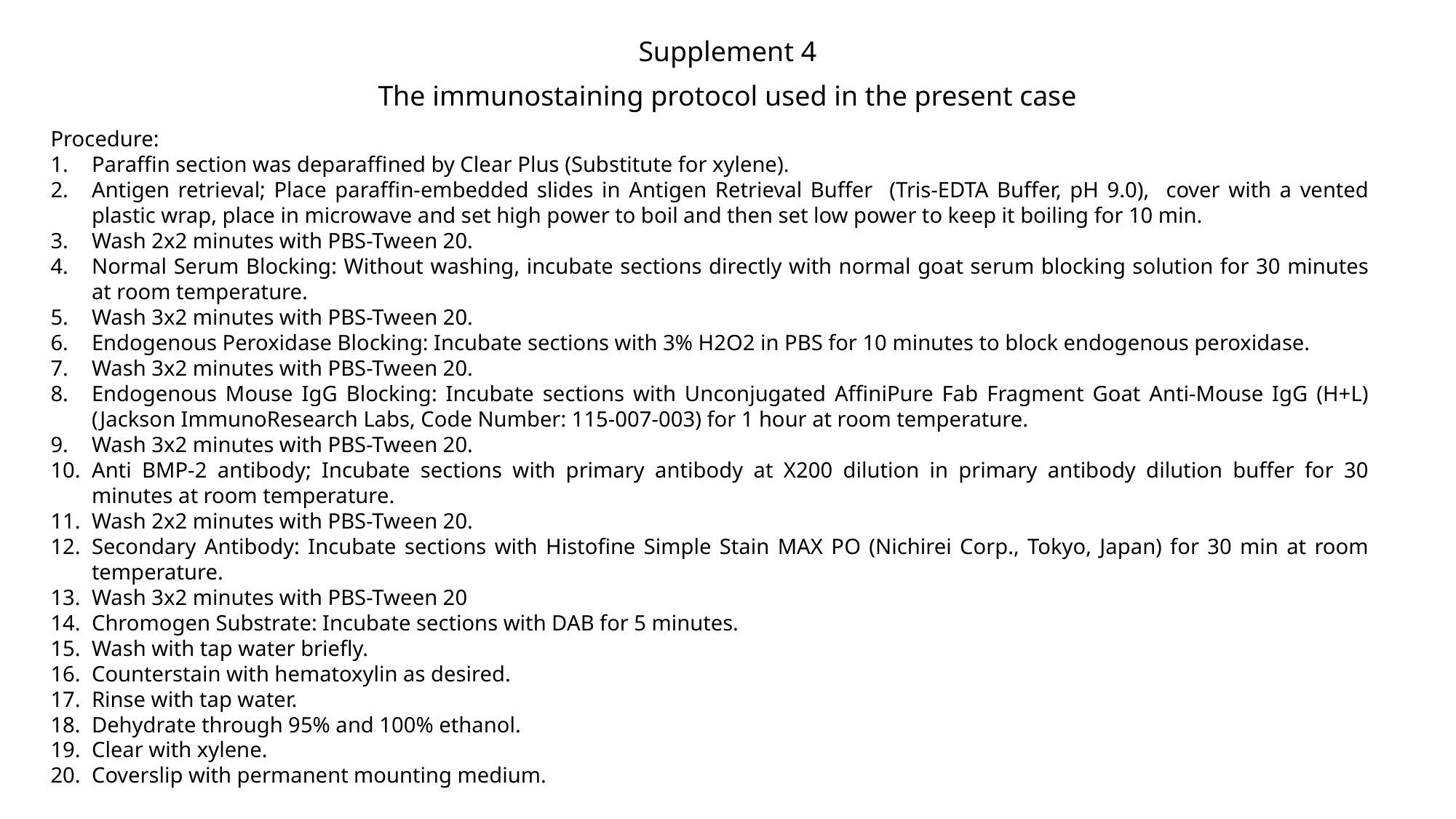

Supplement 4
The immunostaining protocol used in the present case
Procedure:
Paraffin section was deparaffined by Clear Plus (Substitute for xylene).
Antigen retrieval; Place paraffin-embedded slides in Antigen Retrieval Buffer (Tris-EDTA Buffer, pH 9.0), cover with a vented plastic wrap, place in microwave and set high power to boil and then set low power to keep it boiling for 10 min.
Wash 2x2 minutes with PBS-Tween 20.
Normal Serum Blocking: Without washing, incubate sections directly with normal goat serum blocking solution for 30 minutes at room temperature.
Wash 3x2 minutes with PBS-Tween 20.
Endogenous Peroxidase Blocking: Incubate sections with 3% H2O2 in PBS for 10 minutes to block endogenous peroxidase.
Wash 3x2 minutes with PBS-Tween 20.
Endogenous Mouse IgG Blocking: Incubate sections with Unconjugated AffiniPure Fab Fragment Goat Anti-Mouse IgG (H+L) (Jackson ImmunoResearch Labs, Code Number: 115-007-003) for 1 hour at room temperature.
Wash 3x2 minutes with PBS-Tween 20.
Anti BMP-2 antibody; Incubate sections with primary antibody at X200 dilution in primary antibody dilution buffer for 30 minutes at room temperature.
Wash 2x2 minutes with PBS-Tween 20.
Secondary Antibody: Incubate sections with Histofine Simple Stain MAX PO (Nichirei Corp., Tokyo, Japan) for 30 min at room temperature.
Wash 3x2 minutes with PBS-Tween 20
Chromogen Substrate: Incubate sections with DAB for 5 minutes.
Wash with tap water briefly.
Counterstain with hematoxylin as desired.
Rinse with tap water.
Dehydrate through 95% and 100% ethanol.
Clear with xylene.
Coverslip with permanent mounting medium.
